# Adapted Prescription Dose for Monte Carlo Algorithm in Lung SBRT: Clinical Outcome on 205 Patients

**DOI:** 10.1371/journal.pone.0133617

**Published:** 2015-07-24

**Authors:** Jean-Emmanuel Bibault, Xavier Mirabel, Thomas Lacornerie, Emmanuelle Tresch, Nick Reynaert, Eric Lartigau

**Affiliations:** 1 Academic Radiation Oncology Department, Oscar Lambret Comprehensive Cancer Center, 3 rue Frédéric Combemale, Lille, France; 2 Biostatistics Department, Oscar Lambret Comprehensive Cancer Center, 3 rue Frédéric Combemale, Lille, France; 3 Faculty of Medicine, University Lille 2, Lille, France; 4 ONCOLille, maison régionale de la recherche Clinique, Lille, France; Yale School of Public Health, UNITED STATES

## Abstract

**Purpose:**

SBRT is the standard of care for inoperable patients with early-stage lung cancer without lymph node involvement. Excellent local control rates have been reported in a large number of series. However, prescription doses and calculation algorithms vary to a great extent between studies, even if most teams prescribe to the D95 of the PTV. Type A algorithms are known to produce dosimetric discrepancies in heterogeneous tissues such as lungs. This study was performed to present a Monte Carlo (MC) prescription dose for NSCLC adapted to lesion size and location and compare the clinical outcomes of two cohorts of patients treated with a standard prescription dose calculated by a type A algorithm or the proposed MC protocol.

**Patients and Methods:**

Patients were treated from January 2011 to April 2013 with a type B algorithm (MC) prescription with 54 Gy in three fractions for peripheral lesions with a diameter under 30 mm, 60 Gy in 3 fractions for lesions with a diameter over 30 mm, and 55 Gy in five fractions for central lesions. Clinical outcome was compared to a series of 121 patients treated with a type A algorithm (TA) with three fractions of 20 Gy for peripheral lesions and 60 Gy in five fractions for central lesions prescribed to the PTV D95 until January 2011. All treatment plans were recalculated with both algorithms for this study. Spearman’s rank correlation coefficient was calculated for GTV and PTV. Local control, overall survival and toxicity were compared between the two groups.

**Results:**

205 patients with 214 lesions were included in the study. Among these, 93 lesions were treated with MC and 121 were treated with TA. Overall survival rates were 86% and 94% at one and two years, respectively. Local control rates were 79% and 93% at one and two years respectively. There was no significant difference between the two groups for overall survival (p = 0.785) or local control (p = 0.934). Fifty-six patients (27%) developed grade I lung fibrosis without clinical consequences. GTV size was a prognostic factor for overall survival (HR = 1.026, IC95% [1.01–1.041], p<0.001) and total dose was a prognostic factor for local control (HR = 0.924, IC95% [0.870–0.982], p = 0.011). D50 of the GTV calculated with MC correlated poorly with the D95 of the PTV calculated with TA (r = 0.116) for lesions with a diameter of 20 mm or less. For lesions larger than 20 mm, spearman correlation was higher (r = 0.618), but still insufficient.

**Conclusion:**

No difference in local control or overall survival was found between patients treated with a type A or a type B algorithm in our cohort. A size and location adapted GTV-based prescription method could be used with a type B algorithm. External validation of these results is warranted.

## Introduction

Stereotactic Body Radiation Therapy (SBRT) has become a standard modality for curative treatment of inoperable non-small-cell lung cancer with excellent local control rates reported across a large number of studies. Many different treatment regimens have been used, and even within a given regimen, treatment planning and radiation delivery techniques may vary to a great extent [1].

One of the most important sources of differences is the dose calculation algorithm, which is divided in two types. Ray Tracing, a type A algorithm, the delivered dose is calculated by tracing the beam as it travels through the tissue. Tissue heterogeneities are accounted for through a central axis effective depth calculation and there are no corrections for changes in electron transport or lateral scatter disequilibrium. While the results of this type of algorithm is satisfactory for homogeneous structures such as the brain or liver, more robust heterogeneity corrections are required for accurate dose calculations in highly heterogeneous structures such as the lungs, even more so as tissue heterogeneity effects are exacerbated for small field sizes, which are characteristic of most stereotactic radiosurgery systems [2,3]. Type B algorithms take into account electron transport and lateral scatter disequilibrium. Among these, Monte Carlo (MC) is referred to as the ‘gold standard’ for dose calculation [4]. This algorithm considers separately each possible interaction of every particle entering the patient and identifies the material type and density at each voxel. The energy and direction of each photon are randomly generated according to their respective probability distributions. At each voxel the photon travels through, the probability of every possible interaction is assessed and any energy deposition at that voxel is recorded. The properties of any secondary photons or electrons generated in the interaction are saved for later simulation. This process repeats until each photon is absorbed or leaves the model volume (typically several million initial photons).

Studies have shown the importance of the dosimetric calculation algorithm used for treatment planning [5,6]. It is now established that taking into account lateral electron transport in heterogeneous tissue is more accurate in the thoracic region and MC [4]. The clinical implications of MC or type B algorithms have already been illustrated in the literature [7,8]. Protocols for lung SBRT treatment planning now need to be re-evaluated with MC calculations. Our department started a lung SBRT program for inoperable patients with lung cancer in 2007. Until 2011, all patients were treated with a type A algorithm (Ray Tracing) and a standard prescription method. After January 2011, we switched to a type B algorithm (MC), using adapted prescription protocols with the same LINAC (CyberKnife, Accuray Incorporated, Sunnyvale, CA) in order to achieve the same clinical efficacy without increased toxicity.

GTV-based prescription using a MC algorithm has already been described in a previous study [9]. It was determined that, when using a type A algorithm to prescribe the same dose to the PTV, differences between different cases (small vs large, central vs peripheral tumors) were always less than 10% of the prescription dose. But, when using a type B algorithm, prescription to the PTV led to more important discrepancies in the median GTV dose (up to 28%). Directly prescribing to the median GTV dose, using a type B algorithm, considerably reduced this variability.

The aim of the present study was to assess if it is safe to routinely treat patients with a type B algorithm without decreasing the local control or overall survival rates.

## Methods and Materials

Data from inoperable patients with non-small-cell lung cancer treated with SBRT between August 2007 and April 2013 were retrospectively analyzed. Patients with Performance Status under or equal to 2 with T1 or T2 tumors over 15 mm without lymph node or distant metastasis were included in this study. Initial staging included CT-Scan and ^18^F-FDG-PET-Scan. Patients with histologically unverified tumors were treated if the lesion grew on two consecutive CT-Scans (two months apart) and had an FDG uptake. A tumor board discussed each patient’s case with a thoracic surgeon. Previous contra- or ipsilateral lung surgery or radiation therapy was allowed. Patients did not receive chemotherapy before, during, or after treatment until any progression. A thin-sliced CT-scan without contrast was recorded with millimetric slices. The Gross Tumor Volume (GTV) and organs at risk (spinal cord, left and right lung, heart, and esophagus) were contoured. No GTV to CTV margin was added. A 3-mm margin was added to the GTV to create the planning target volume (PTV). Patients were treated with tumor tracking (with or without fiducial placement) or without tracking after an ITV was contoured on a 4D CT-Scan.

Treatments were planned on Accuray's Multiplan software. The prescription dose was calculated with the Monte Carlo algorithm with a 2% uncertainty on a 1x1x1-mm resolution with Gaussian smoothing. The following prescription method was adopted. 50% of the GTV was to receive the dose as follows: 54 Gy in three fractions for peripheral lesions with a diameter under 30 mm, 60 Gy in three fractions for lesions with a diameter over 30 mm, and 55 Gy in five fractions for central lesions. Treatment fractions were delivered every other day with a 6-MV CyberKnife linear accelerator. Image guidance was performed with stereoscopic imaging between and during treatment sessions.

The primary endpoint was local control of the irradiated lesion treated with the MC protocol compared to a cohort of patients treated with a type A algorithm (Ray Tracing), with a protocol that mandated that 95% of the PTV receive the dose as follows: 60 Gy in three fractions for peripheral lesions and 60 Gy in five fractions for central lesions. Patients were routinely followed up with CT scans every 3 months for the first 2 years, every 6 months for 3 years, and then annually after 5 years. Specific therapeutic response criteria for lung SBRT were used [10]. Positron emission tomography/CT was used to confirm recurrence no less than 6 months after treatment. Toxicity was assessed with the CTCAE v4.0 scale [11].

All plans were recalculated with TA and MC with identical monitor units per beam to assess the correlation between the two algorithms. We estimated the average difference between the calculated TA dose and the dose actually delivered: dose to 95% of the PTV (D95PTV), to 98% (D98GTV) and 50% (D50GTV) of the GTV, average dose received by the lungs and V20 lungs calculated with MC were chosen as representative dosimetric indices for comparison.

Statistical analysis was performed with Stata v11.2 (StataCorp. 2009. Stata Statistical Software: Release 11. College Station, TX: StataCorp LP). Pearson’s chi-square tests were used to compare groups when events frequencies were more than 5. Fisher exact tests were used when events frequencies were less than 5. All tests were performed with significance level of 0.05. Pearson coefficients were calculated to assess the correlation between the two algorithms. Influence of age, sex, comorbities, medical history, and disease stage was examined with univariate logistic regression. Multivariate logistic regression was to be performed if more than one factor was associated with one of the outcomes on interest with a p value of less than 0.05. Local control and overall survival were assessed with the Kaplan-Meier method.

### Ethics

This study was approved by the internal ethic board of our institution (Clinical Trial Commission; “Commission interne des études cliniques”). Our institutional review board waived the need for written informed consent from the participants. French laws (Data, data-collection and freedom law, January, 6th 1978) state that in case of single-centre, retrospective study based on already recorded and stored data, there is no need of specific written informed consent. All patients have been orally informed about the potential use of their collected data for future research. Agreement N1034071 was obtained from the “National Commission about Data-collection and Freedom” (“Commission Nationale Informatique et Liberte”) for the conduct of this study

## Results

We treated 205 patients with 214 lesions. Ninety three lesions were treated with the MC protocol (43.4%) and were compared to 121 lesions (56.5%) treated with a standard Type A algorithm (TA). 84.4% (n = 173) were men. Median age was 70 years. Of the inoperable patients, 79.5% had insufficient lung function (n = 163) and 8.3% had heart disease (n = 17). For 75.6% of the patients, this was their first treatment for non-small-cell lung cancer (n = 155). Among the other patients, 14.1% had already undergone a lobectomy for a previous lung cancer (n = 29), 3.9% a pneumonectomy (n = 29), and 6.3% SBRT (n = 13). Seven patients had a pacemaker. Patients’ characteristics were well balanced between the two groups (Table 1). Histologically identified lesions comprised 47.7% of all (n = 102). Median tumor diameter was 22 mm (range, 15–60 mm). Lesions’ sizes and locations were also well balanced between the two groups (Table 2). We treated 55.6% of the lesions without tracking (n = 119). Median GTV was 5418 mm^3^. Median number of beams was 58 (range, 20–207 beams) with a median 41 nodes (range, 17–88 nodes). Median fraction duration was 37 minutes (range, 15–134 minutes). Median V20 to the lungs was 2.5 Gy (range, 0–25 Gy). Average dose to both lung (GTV excluded) was 0.3 Gy (range, 0.05–1.22 Gy) in TA. Technical data are shown in Table 3.

**Table 1:** Baseline patient characteristics.

Patients characteristics (N = 205)	Ray Tracing		Monte Carlo		p value
	N	%	N	%	
**Sex**					0.38
Male	101	86.3%	72	81.8%	
Female	16	13.7%	16	18.2%	
**Age (years)**					0.71
Median (range)	69	(49–92)	70.5	(46–87)	
**Reason for Inoperability**					0.20
Respiratory failure	96	82.1%	67	76.1%	
Heart failure	6	5.1%	11	12.5%	
Refusal	6	5.1%	6	6.8%	
Cirrhosis	4	3.4%	1	1.1%	
Other	5	4.4%	3	3.4%	
**Previous treatment**					0.18
None	88	75.2%	67	76.1%	
Lobectomy	13	11.1%	16	18.2%	
SBRT	10	8.5%	3	3.4%	
Pneumonectomy	6	5.1%	2	2.3%	

**Table 2:** Baseline lesion characteristics. Abbreviations: LLL = left lower lobe; LUL = left upper lobe; RLL = right lower lobe; RML = right middle lobe; RUL = right upper lobe.

Lesion characteristics (N = 214)	Ray Tracing		Monte Carlo		p value
	N	%	N	%	
**Tumor classification**					0.95
T1a	50	42.0%	36	38.7%	
T1b	36	30.3%	31	33.3%	
T2a	30	25.2%	24	25.8%	
T2b	3	2.5%	2	2.2%	
**Tumor localization**					0.99
Central	65	53.7%	50	53.8%	
Peripheral	56	46.3%	43	46.2%	
**Treatment lobe**					0.35
RUL	46	38.0%	29	31.2%	
RLL	16	13.2%	21	22.6%	
RML	7	5.8%	3	3.2%	
LUL	38	31.4%	27	29.0%	
LLL	14	11.6%	13	14.0%	

**Table 3:** Technical characteristics of treatments.

Characteristics (N = 214)	Median	Min	Max	Median	Min	Max	p value
**D95PTV Ray Tracing** (Gy)	60.06	34.85	87.26	57.205	34.84	73.47	0.002
**D98GTV Monte Carlo** (Gy)	50.45	34.40	67.16	49.03	30.11	60.83	0.24
**D50GTV Monte Carlo** (Gy)	57.015	36.57	64.08	54.55	32.62	66.96	0.019
**V20Gy lung** (%)	2.6	0.2	14.3	2.5	0	24.9	0.87
**Average lung dose** (Gy)	0.301	0.58	8.54	2.84	0.52	12.18	0.69

**Fig 1:**
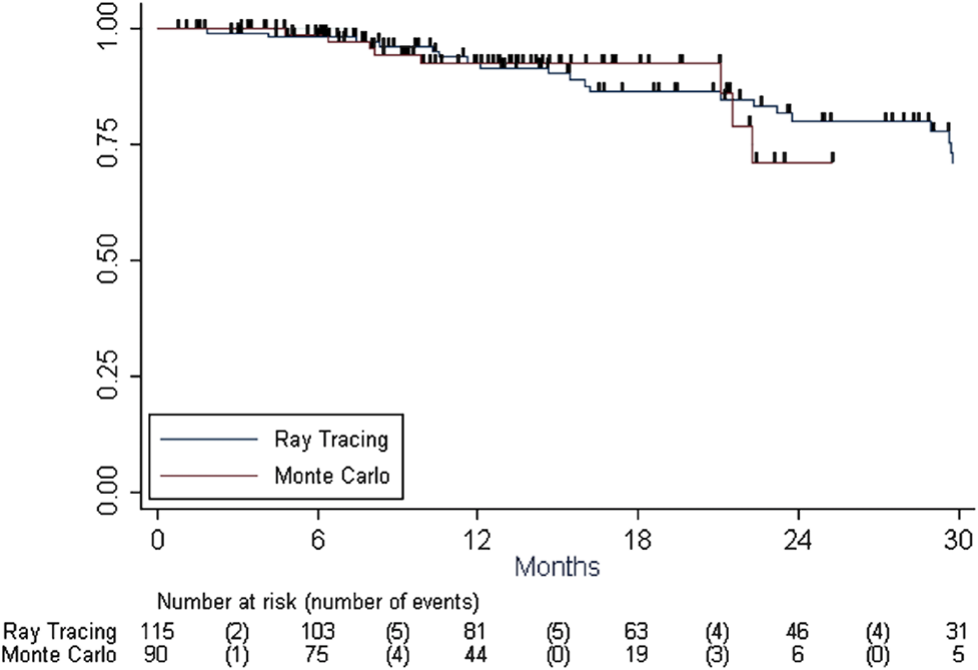
Kaplan-Meier curves for local control.

Median follow-up was 15 months for the MC cohort (range, 3–40 months), vs 24 months for the TA cohort (range, 3–55 months). Fifty-one patients (25%) had a local (n = 8) or distant (n = 43) recurrence. Twelve percent of the patients (n = 24) died during follow-up, 50% of them from cancer. Local control rates at 1 and 2 years were 93% (95% CI = 87.3–95.7%) and 79% (95% CI = 69.2–85.7%), respectively. Overall survival rates at 1 and 2 years were 94.3% (95% CI = 89.2–97.0%) and 86.2% (95% CI = 77.8–91.6%), respectively. The algorithm used for treatment planning was not a prognostic factor for local control (p = 0.740, Fig 1) or overall survival (p = 0.785). Tumor diameter (HR = 1.160, p = 0.006, IC95% [1.0427–1.2895]) and total dose (HR = 0.924, p = 0.011, IC95% [0.870–0.982]) were the only significant prognostic factors for local control. Seventy-three patients had toxicities: 6.8% (n = 14) had radiation pneumonitis and 27% (n = 56) had lung fibrosis (lung scar six months after treatment). Two patients had a rib fracture. There was no difference in toxicity between the two groups (p>0.05).

Correlations between D95PTV, D98GTV, and D50GTV for all 214 lesions were assessed using the Spearman method because their distributions were not normal. The value of the correlation coefficient represents the strength of the association between two variables. With Monte Carlo, D98GTV and D50GTV were highly correlated, as expected (r = 0.858). When we compared D95PTV in TA with D50GTV in MC, the correlation coefficient was 0.216, meaning that D95PTV calculated with TA did not correctly reflect the dose actually received by the tumor.

**Fig 2:**
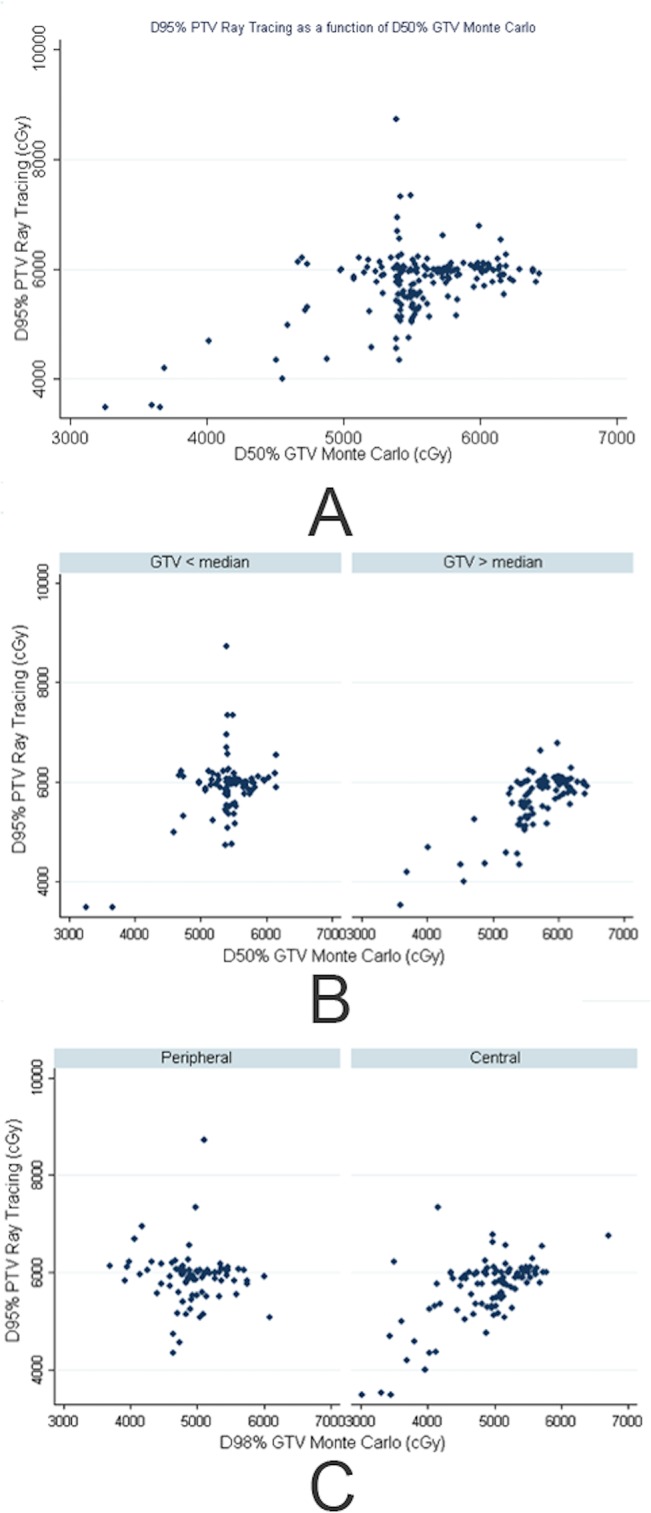
A: D95PTV in TA as a function of D50GTV in MC B: D95PTV in TA as a function of D50GTV in MC according to GTV size–C: D95PTV in TA as a function of D50GTV in MC according to lesion localization.

For smaller GTVs (GTV < 20 cm^3^ in our cohort), the correlation was even worse (r = 0.116). Fig 2 shows D95PTV in TA as a function of D50GTV in MC for all lesions (A), for lesions according to the size of the PTV (PTV < median r = 0.116 vs PTV > median r = 0.618) (B), and according to their location (peripheral, r = -0.064 vs central, r = 0.529) (C).

## Discussion

Discrepancies in dose calculation may lower the probability of tumor control or increase toxicity to the surrounding lung tissue and impair our ability to report and compare different therapeutic protocols. It is now a well-known fact that type A algorithms (including Ray Tracing) overestimate the dose to the Planning Target Volume when used for lung SBRT calculation because it does not take into account the lack of lateral electronic equilibrium and changes of scattered dose and simply considers the decreased attenuation of the primary photon beam in low-density tissue [12–15]. Type B algorithms, such as Monte Carlo, take into account these effects and are now considered as the Gold Standard for dose calculation [16–20]. Because calculation times have been drastically reduced on recent workstations, MC can be used routinely in radiation oncology departments. With MC, each photon and electron is calculated individually. The differences between the two algorithms are higher on the edge of the target because the dose in this area is dependent on the lack of electronic equilibrium. For smaller lesions and treatment beams, bigger discrepancies are observed [3]. While these differences are well described in several studies [12–15], it is impossible to simply convert treatment protocols from a type A to a type B algorithm because dose differences depend on several factors such as beam size, rearrangement, energy, tumor size and location and lung density. Three propositions can be found in the literature. One of them can be found in the ROSEL study, a phase III randomized trial designed to compare lung SBRT to surgery [21]. The authors recommended three fractions of 20 Gy when calculations were based on Type A algorithms and three fractions of 18 Gy when treatment planning was based on Type B algorithms. Another study made similar recommendations with three fractions of 18–19 Gy when converting from dose calculation based on unit density to ‘‘type b” dose calculation [5]. These two studies did not provide a recommendation based on tumor size, even though it was already known that could affect the dose actually received by the tumor. In 2010, a third study published by van der Voort van Zyp et al. proposed an MC prescription dose according to tumor size and location [22]. The authors showed that for central lesions, the MC dose was reduced less than that for peripheral tumors (4–5% vs 3–5%), as in our study. The authors also noted that dose constraints to OARs could be easily converted because the correlation between the type A and type B calculation was high for OARs (R^2^ = 0.98–0.99). We did not find any significant differences in V20 or average dose to the lungs between the two cohorts in our study either, which was expected, even though we did find a poor correlation between D95PTV TA and D50GTV MC (p = 0.216) and a significant difference in calculated doses (p = 0.019; Table 3).

A more recent study compared two cohorts of patients treated with Pencil Beam (PB type A) or Collapsed Cone Convolution (CCC–type B) algorithms [8]. Two hundred and one patients were treated to 50 Gy in five fractions of 10 Gy each. The prescription mandated that 95% of the PTV receive the prescribed dose. One hundred and sixteen patients were planned with the PB algorithm on a Novalis with ExacTrac (Brainlab AG, Feldkirchen, Germany) unit and 85 with the CCC algorithm on a Varian Linac, either Trilogy or TrueBeam (Varian Medical Systems, Palo Alto, CA, USA). With a primary objective local/marginal control, the authors found a statistical difference in recurrence rates between the two groups in favor of the patients treated with CCC (HR = 3.4, 95% IC = 1.18–9.83). The same prescription protocols were applied to all patients, without taking into account lesion size or location. The authors of the study addressed several biases. The two groups of patients were treated with two very different techniques: while the patients treated with PB had stereoscopic radiographs on the Novalis, patients treated with CCC had CBCT guidance on the Trilogy or TrueBeam. In the same manner, patients treated with PB were treated with a 3D technique and 10 MV beams, while the other patients underwent a 15 MV VMAT technique, meaning that dose conformality could have been very different between the two groups. Finally, the fractionation regimen’s biologically equivalent dose was barely sufficient for tumor control [23,24]. With even a small dosimetric difference between the PB and CCC groups, local control was consequently different. In that regard, findings of this study only confirm and underline the need to adapt prescription models to type B algorithms in lung SBRT, not for OARs but for tumor control. The present study is the first to assess a tumor-size- and location-adapted dose prescription protocol on a large cohort of 205 patients.

We showed on a large cohort that prescribing to the D95PTV while using TA does not faithfully reflect the dose actually received by the tumor, especially when the lesion is peripheral and small. Prescribing to the D50GTV with MC is feasible and provides excellent results in both local control and overall survival. One could argue that there is no need to adapt the prescription because the reported local control rates were already excellent with a type A algorithm, but we believe the minimal sufficient dose should be used, especially for inoperable patients with lung disease. The main limitations of this study are its retrospective nature and the difference in follow-up times between the two groups. But other major confounding factors found in other studies, such as heterogeneous treatment techniques or prescriptions are not a concern in the presented work. The results of this study should be confirmed in a prospective and multicentric manner to further validate the use of a GTV-based type B algorithm prescription.

## Conclusion

In our series, using of a type B algorithm did not alter the local control and overall survival, when compared to a cohort of patients treated with a type A algorithm in our center. A type B algorithm should be preferred for lung SBRT dose calculation to limit dose differences between cases. External validation of this prescription method is warranted.
